# Factors associated with food safety knowledge and practices among meat handlers in Bangladesh: a cross-sectional study

**DOI:** 10.1186/s12199-021-01004-5

**Published:** 2021-08-28

**Authors:** Md. Hasan Al Banna, Tasnim Rahman Disu, Satyajit Kundu, Bright Opoku Ahinkorah, Keith Brazendale, Abdul-Aziz Seidu, Joshua Okyere, Nahidur Rahman, Shuvajit Mondal, Bidyut Matubber, Md Shafiqul Islam Khan

**Affiliations:** 1 Department of Food Microbiology, Faculty of Nutrition and Food Science, Patuakhali Science and Technology University, Patuakhali, 8602 Bangladesh; 2 Medical Officer, Institute of Public Health Nutrition, Mohakhali, Dhaka, 1212 Bangladesh; 3 School of Public Health, Southeast University, Nanjing, 210009 China; 4 School of Public Health, Faculty of Health, University of Technology Sydney, Sydney, Australia; 5 Department of Health Sciences, University of Central Florida, Orlando, USA; 6 Department of Population and Health, University of Cape Coast, Cape Coast, Ghana; 7 College of Public Health, Medical and Veterinary Services, James Cook University, Brisbane, Australia; 8 Department of Food Processing and Engineering, Chattogram Veterinary and Animal Sciences University, Chattogram, 4225 Bangladesh; 9 Faculty of Nutrition and Food Science, Patuakhali Science and Technology University, Patuakhali, 8602 Bangladesh; 10 Department of Microbiology and Public Health, Khulna Agricultural University, Khulna, Bangladesh

**Keywords:** Food safety knowledge, Food safety practices, Meat handlers, Bangladesh

## Abstract

**Background:**

Food handlers can play a vital role into reducing foodborne diseases by adopting appropriate food handling and sanitation practices in working plants. This study aimed to assess the factors associated with food safety knowledge and practices among meat handlers who work at butcher shops in Bangladesh.

**Methods:**

A cross-sectional study was conducted among 300 meat handlers from January to March, 2021. Data were collected through in-person interviews using a structured questionnaire. The questionnaire consisted of three parts; socio-demographic characteristics, assessments of food safety knowledge, and food safety practices. A multiple logistic regression model was used to identify the factors associated with food safety knowledge and practices.

**Results:**

Only 20% [95% confidence interval, (CI) 15.7–24.7] and 16.3% (95% CI 12.3–20.7) of the respondents demonstrated good levels of food safety knowledge and practices, respectively. The factors associated with good levels of food safety knowledge were: having a higher secondary education [adjusted odds ratio (AOR) = 4.57, 95% CI 1.11–18.76], income above 25,000 BDT/month (AOR = 10.52, 95% CI 3.43–32.26), work experience of > 10 years (AOR = 9.31, 95% CI 1.92–45.09), ≥ 8 h per day of work (AOR = 6.14, 95% CI 2.69–13.10), employed on a daily basis (AOR = 4.05, 95% CI 1.16–14.14), and having food safety training (AOR = 8.98 95% CI 2.16–37.32). Good food safety knowledge (AOR = 5.68, 95% CI 2.33–13.87) and working ≥ 8 h per day (AOR = 8.44, 95% CI 3.11–22.91) were significantly associated with a good level of food safety practice.

**Conclusions:**

Poor knowledge and practices regarding food safety were found among Bangladeshi meat handlers. Findings may help public health professionals and practitioners develop targeted strategies to improve food safety knowledge and practices among this population. Such strategies may include education and sensitization on good food safety practices.

## Background

Foodborne diseases have become a public health concern across the world and have been linked to poor food handling and sanitation practices among food handlers [1, 2]. As such, promoting the hygienic practices in the area of food handling is deemed a plausible and potentially effective strategy to protect consumers from public health risks [3]. Poor food handling practices and hygiene accounts for a substantial proportion of foodborne disease outbreaks within the population [4, 5]. Furthermore, evidence estimates that more than 200 types of diseases can be spread through food [6].

In Bangladesh, the occurrence of foodborne diseases and other food safety hazards is substantially high due to the densely populated nature, underdeveloped infrastructure and poor water, sanitation, and hygiene (WASH) conditions [7]. Approximately 30 million people experience one form of foodborne disease each year in Bangladesh [8]. However, there is no data on foodborne illness or food poisoning directly attributable to meat handlers or meat in Bangladesh. Animal-source foods, such as meats, fish, and their products are highly susceptible to foodborne diseases due to the food being an ideal growth medium for pathogens and other possible contaminants [9]. Previous Bangladeshi studies [10–12] found that meat samples contain pathogenic microorganisms such as *Escherichia coli, Salmonella* sp., and *Staphylococcus aureus*, which have the potential to cause food poisoning in consumer.

Collectively, diarrheal diseases are the most common foodborne diseases in Bangladesh, followed by enteric fever and hepatitis [13]. Evidence from a report by the Dhaka-based Institute of Epidemiology, Disease Control and Research (IEDCR) showed that acute watery diarrhea is the most prevalent outcome as a result of food poisoning in the country, with around 0.28 million cases in 2015 [14, 15]. Another two common foodborne illnesses, enteric fever and hepatitis, occur in approximately 30,000 and 500 people per year, respectively [14, 15]. Consequently, there may be additional effects of contracting a foodborne disease that may impact an individuals’ health and well-being (e.g., long-lasting disability, death) or pose a financial burden [16]. In many low-income and middle-income countries including Bangladesh, foodborne disease outbreaks may impair socio-economic development by straining the local health care systems and harming the national economy (e.g., trade and tourism), and at the individual level, hamper the productivity of the individual [17]. A Bangladeshi review on food safety issues reported a high incidence of disease-causing microbes in street-vended food, adulterated food, and food processed with contaminated water, in addition to unhygienic and unsanitary conditions [7]. However, Bangladesh’s current food safety system such as the testing equipment, laboratories, and health and safety evaluation systems are inadequate to detect and control these foodborne pathogens and food safety hazards [15].

One area of food processing that is susceptible for food contamination and spread of foodborne diseases is within the meat handling and slaughtering sectors. According to Nyamakwere et al. [18], the meat handling section in food processing plants is characterized by intensive handling and slaughtering of carcasses in a multi-step process. Therefore, poor hygienic practices (e.g., non-use of gloves, protective clothing, and disinfectants) in meat handling facilities can lead to food contamination and the spread of foodborne diseases [19].

Globally, Hazard Analysis and Critical Control Point (HACCP) is recognized as an important food safety system that eliminates likely chemical, biological, and physical hazards [20]. Countries such as the USA [21] and Serbia [22] have mandatory HACCP requirements. However, in Bangladesh, the development of HACCP has been slow; primarily due to the high start-up cost of implementing nationwide, mandatory HACCP systems [23]. Bangladesh Standards and Testing Institution (BSTI) has developed approximately 600 agriculture and food standards, including the guidelines for the application of the HACCP system (examples of food standards include BDS CAC GL 18:1998 which deals with HACCP system) [24, 25]. Generally, in Bangladesh, food processing sectors (e.g., seafood) representing large companies that export foods (e.g., frozen fish, shrimp) are better equipped to comply with the food safety certifications, including International Standard Organization (ISO) 22000 and HACCP certifications [15].

However, several meat processing units and abattoirs in Bangladesh operate under sub-optimal quality control systems [26]. Alam et al. [27] report that meat handlers in Bangladesh are mostly found in urban areas compared to semi-urban areas; are mostly middle-aged persons (31–40 years); and tend to have completed primary school, with approximately 15% and 5% completing secondary and tertiary school, respectively. Moreover, all the Bangladeshi meat handlers working at butcher shops are male [27], as traditionally, raising children and maintaining the household are still female-dominated activities in Bangladesh. Thus, women do not have much interest, nor priority for, working in the food service industry given these challenges and the additional risks of working at butcher shops, slaughter houses and fish processing markets (e.g., injuries from live animals, knife cuts, cuts from bones).

Evidence from Kenya [3], Portugal [5], South Africa [18], and Saudi Arabia [28] have concluded that knowledge about food safety and appropriate food handling practices translated into better food safety practices among meat handlers. However, there is also evidence of variations in the level of food safety knowledge and practices among meat handlers. For example, Nyamakwere et al. [18] reported that educational level and professional training of meat handlers was significantly associated with their level of knowledge and food safety practices. Similar findings were reported by Al-Shabib and Husain [28]. Food handler training found to be effective in improving food safety knowledge and practices, maintaining sanitary and hygienic parameters and microbial quality of the products [29, 30]. Though in Bangladesh, science-based food safety management systems are emphasized in the Food Safety Act-2013, there are gaps in regular food safety training of food handlers, particularly for under-supervised meat handlers from butcher shops. Several governmental and non-governmental organizations have arranged food safety training for Bangladeshi food handlers, but the activities were not executed in the long-term and on regular basis. To date, there has been no empirical study that has focused on the factors associated with food safety knowledge and practices among meat handlers in Bangladesh. This is of paramount importance, given the high number of Bangladeshi people that contract a foodborne disease each year. This study aimed to assess the factors associated with food safety knowledge and practices among meat handlers in butcher shops in Bangladesh.

## Methods

### Study sites and period

The current study was carried out in four districts situated at the southern coastal regions of Bangladesh from January to March 2021. The coastal area of Bangladesh consisted of 19 districts accounting for 32% of the land area and 25.7% of the total population of Bangladesh [31]. Geographic locations made this area vulnerable to different devastating natural disasters (e.g., floods, cyclones, tidal surges, river erosion) which have had an adverse impact on the socio-economic, water, sanitation, and hygiene problems (such as foodborne diseases like diarrhea) of populations living in this part of the country [32–34]. Thus, four coastal districts namely, Barishal, Patuakhali, Pirojpur, and Chattogram were randomly selected for the study.

### Study design, participants, and sampling

A cross-sectional study was conducted among 300 meat handlers working at butcher shops from the selected areas (*n* = 4) of Bangladesh. A systematic random sampling technique was employed to select each butcher shop followed by an equal allocation of participants where there was more than one individual working in the butcher shop. Initially, research staff visited the respective authorities of the selected areas (city-corporation or municipality) and inquired about a documented list of locally operated butcher shops. Unfortunately, there was no official list but the local authorities provided an idea of the location of local butcher markets, and the research staff noted the location names and visited these areas accordingly. Seventy-five butcher shops from each of the four districts were selected randomly from the main butcher market areas. The butcher shops were small and had an average of three to four workers per shop. Participants were considered eligible for the study based on the following criteria: (i) participants who have direct contact with meat or meat handling surfaces, (ii) having at least 6 months working experience in butcher shops, and (iii) participants free from any disability and illness. All participants were enrolled by lottery techniques from each butcher shop carried out by research staff. The name of each worker in a particular butcher shop was written on a piece of paper, folded, and entered into a container with other pieces of folded paper with butcher shop worker names. A research staff member picked one name from the container and assessed the individual for study eligibility.

### Sample size determination

A sample size of 384 was calculated using Cochran’s formula [35] by considering 50% prevalence of expected food safety knowledge and practice among meat handler as similar studies were lacking in Bangladesh. A 95% confidence interval (CI) and 5% margin of error between the sample and the underlying population was employed. Subsequently, we used a modified Cochran’s formula [35] for calculating adjusted sample size in a small population (assuming 1000 meat handlers work at butcher shops in the selected areas), which gave us a minimum sample size of 277. In anticipation of any missing data or incomplete surveys, the research staff enrolled more participants, and the final sample included in this study was 300.

### Data collection procedures

Prior to data collection, the research protocols were reviewed and approved by the Research Ethical Committee (REC) of Department of Food Microbiology, Patuakhali Science and Technology University, Bangladesh (approval number: FMB:15/12/2020:04). Data collectors explained the purposes of the study to all participants and asked those willing to participate to sign a consent form or obtained verbal consent from those who were illiterate. Data were gathered by in-persons interviews of a structured questionnaire, which was based on previous literature [36, 37]. The English-version of the questionnaire was translated into the local language (Bengali) at the time of data collection. First translation of the questionnaire from English to Bengali was conducted by a bilingual translator, which was cross-checked by an independent bilingual research staff member. Further, a back-translation of the questionnaire was conducted by another, independent bilingual research staff member to check for consistencies and to avoid any bias in the questionnaire. The pre-test of the questionnaire was done among a randomly-selected small group of meat handlers (*n* = 15) to verify the applicability and clarity of the questionnaires, and the time needed for each interview. Anonymity of participants’ responses was ensured through the coding of questionnaires. Each interview took 15–20 min to complete at the butcher shop of participant/workers.

### Study variables and measures

A questionnaire was adapted from previous studies [36, 37] with certain questions modified based on the specific socio-demographic status of the target sample (i.e., meat handlers who work at butcher shops) and the differing perspectives of persons living in Bangladesh versus the other countries where previous studies had taken place. Based on the pre-testing of the questionnaire, modifications were made to several items pertaining to food safety knowledge as test respondents noted that aspects of these questions or statements were unclear. For example, we added an item, “Anthrax can be transmitted by food” instead of “AIDS can be transmitted by food”. The questionnaire included a total of 43 questions with three sections as follows: (i) socio-demographic information, (ii) assessments of food safety knowledge and (iii) assessments of food safety practice.

In the first section, socio-demographic characteristics such as, gender, age, education level, years of experience, working hour per day, field of duty, income, employment status, having health certificate and attending food safety training of the respondents were included.

For assessing food safety knowledge, a set of 18 close-ended questions with three possible answers (such as “true,” “false,” and “do not know”) were used. The food safety knowledge questions included information on personal and food hygiene, cross-contamination of food, high-risk groups for food poisoning, and specific foodborne diseases and pathogens. To reduce the possibility of respondents selecting the correct answer by chance, the multiple-choice answers included the option “do not know.” One point was assigned for each “true” answer, with the other two answers (“false” and “do not know”) assigned zero score. The food safety knowledge score ranged between 0 and 18, and scores 9 or above was considered a good level of knowledge, and a score of below 9 was indicated as a poor level of knowledge [37].

The last section of the questionnaire dealt with food safety practices of the respondents emphasizing issues of personal hygiene, hand washing practices, and practices against food borne diseases and cross-contamination. A set of 15 questions was provided and the level of practices were assessed using a five-point ordinal scale (never = 0, rarely = 1, sometimes = 2, often = 3 to always = 4). For four questions, reversed scoring was employed (items 2, 3, 12, and 15). To avoid the possibility of respondents picking the correct answer by chance, the option “sometimes” was included. The score ranged between 0 and 60, and scores below 30 were recognized as poor practices, and scores 30 or above were considered as a good level of practice [37]

### Statistical analysis

Data were analyzed by Statistical Package for the Social Sciences (SPSS) software (version 23.0). Descriptive statistics (e.g., response frequencies/percentage, means and standard deviations) were used to summarize variables of interest. Analytical statistics including bivariate analyses and multiple logistic regression models was used to identify factors associated with food safety knowledge and practices of meat handlers. All socio-demographic variables except gender and health certificates (excluded due to lack of variation of categories) were included in both univariable (unadjusted) and multivariable (adjusted) logistic regression models. Multicollinearity among the independent variables was checked using variance inflation factor (VIF) and tolerance. The mean VIF for adjusted model of Table 4 was 1.463 (Min VIF = 1.056, Max VIF = 2.432), while the mean VIF for adjusted model of Table 5 was 1.374 (Min VIF = 1.116, Max VIF = 1.751). Previous studies reported that a mean VIF of less than 10 is acceptable [38, 39]. The strength of association between independent variables (such as age, education level, and income) and dependent variables (level of food safety knowledge and practice) was assessed by odds ratios with 95% confidence interval (CI). And a *p* value of less than 0.05 was considered statistically significant.

## Results

Socio-demographic profiles of the respondents are presented in Table 1. Of the 300 meat handlers who participated in this study, all were males. The mean age of respondents was 31 years (SD = 9.4), ranging between 16 and 67 years. Nearly one-fourth of the respondents (22.0%) had no formal education. More than half (53.3%) of respondents had worked for more than 5 years, but very few respondents had food safety training (5.3%) and a health certificate (1.0%) (Table 1).

**Table 1:** Socio-demographic characteristics of study participants (*N* = 300)

Variables	Categories	Frequency	Percentage
**Gender**	Male	300	100
**Age (years)** Mean ± SD 31 ± 9.4	< 20	24	8.0
	21–30	114	38.0
	31–40	118	39.3
	> 40	44	14.7
**Field of duty**	Butcher	160	53.3
	Helper	140	46.7
**Education level**	No formal education	66	22.0
	Primary	117	39.0
	Secondary	79	26.3
	Higher secondary	38	12.7
**Monthly earned, BDT**^a^	< 10,000	59	19.7
	10,001–15,000	96	32.0
	15,001–20,000	60	20.0
	20,001–25,000	39	13.0
	> 25,000	46	15.3
**Work experience**	< 5 years	140	46.7
	5–10 years	90	30.0
	> 10 years	70	23.3
**Working hour/day**	< 8 h	163	54.3
	≥ 8 h	137	45.7
**Employment status**	Permanent	169	56.3
	Daily	71	23.7
	Contract	60	20.0
**Food safety training**	Yes	16	5.3
	No	284	94.7
**Health certificate**	Yes	3	1.0
	No	297	99.0

^a^10,000 Bangladeshi Taka (BDT) equals to 125 USD

### Food safety knowledge and its associated factors among meat handlers

Assessment of food safety knowledge of meat handlers is summarized in Table 2. The majority of the respondents reported high knowledge of general personal hygiene and sanitary practices in the workplace such as washing hands before work (95.7%), using gloves, caps and aprons (89.7%), and proper cleaning of the instruments (89.3%). Close to half of the respondents (44.3%) either did not know or wrongly answered about high-risk groups for food poisoning. Overall, most of the respondents reported lower knowledge regarding selected foodborne diseases and foodborne pathogens (Table 2).

**Table 2:** Summary of questions and responses for assessment of food safety knowledge of meat handlers working at butcher shops in Bangladesh (*N* = 300)

Statements	Responses, *n* (%)		
	True	False	Don’t know
Washing hands before work reduces the risk of food contamination.	287 (95.7)	0 (0)	13 (4.3)
Using gloves during work reduces the risk of food contamination.	269 (89.7)	0 (0)	31 (10.3)
Proper cleaning and handling of instruments reduces the risk of food contamination.	268 (89.3)	0 (0)	32 (10.7)
Washing and disinfecting working surfaces and tools are important practices for safely handling meat.	200 (66.7)	0 (0)	100 (33.3)
Eating and drinking in the work place increases the risk of food contamination.	100 (33.3)	37 (12.4)	163 (54.3)
All persons, including children, adults, pregnant women and the elderly are at equal risk for food poisoning.	167 (55.7)	21 (7.0)	112 (37.3)
Typhoid can be transmitted by food.	136 (45.3)	10 (3.4)	154 (51.3)
Jaundice can be transmitted by food.	136 (45.3)	26 (8.7)	138 (46.0)
Diarrhea can be transmitted by food.	183 (61.0)	28 (9.3)	89 (29.7)
Brucellosis can be transmitted by food.	3 (1.0)	18 (6.0)	279 (93.0)
Anthrax can be transmitted by food.	42 (14.0)	18 (6.0)	240 (80)
*E. coli* is a foodborne pathogen.	19 (6.3)	6 (2.0)	275 (91.7)
Salmonella is among the foodborne pathogens	2 (0.7)	0 (0)	298 (99.3)
Staphylococcus is among the foodborne pathogens.	3 (1.0)	0 (0)	297 (99.0)
*Clostridium botulinum* is among the foodborne pathogens.	3 (1.0)	0 (0)	297 (99.0)
During infectious disease of skin and eye, it is necessary to take leave from work.	103 (34.3)	68 (22.7)	129 (43.0)
Cross contamination is when microorganisms from a contaminated food are transferred by the food handler’s hands or utensils to another person.	136 (45.3)	30 (10.0)	134 (44.7)
The correct temperature for storing perishable foods is 5^0^C.	5 (1.7)	0 (0)	295 (98.3)

The mean score for food safety knowledge was 7.0 (SD = 1.9, range 2–13) on a scale of 18.0. Approximately 20% (95% CI 15.7–24.7) of the respondents had good food safety knowledge. Chi-square test found that monthly family income (*p* < 0.001), working hours per day (*p* < 0.001), and food safety training (*p* < 0.001) were significantly associated with food safety knowledge (Table 4).

Table 4 reports the findings from multiple logistic regression predicting the factors associated with good levels of food safety knowledge among study participants. The adjusted regression model showed that respondents who had higher secondary education [adjusted odds ratio (AOR) = 4.57, 95% CI 1.11–18.76], earned above 25,000 Bangladeshi taka (BDT)/month (AOR = 10.52, 95% CI 3.43–32.26), had a work experience of > 10 years (AOR = 9.31, 95% CI 1.92–45.09), worked for ≥ 8 h per day (AOR = 6.14, 95% CI 2.69–13.10), and had food safety training (AOR = 8.98 95% CI 2.16–37.32) were more likely to have a good level of food safety knowledge compared to their counterparts. Moreover, respondents who were employed on a daily basis (AOR = 4.05, 95% CI 1.16–14.14) had higher odds of having a good level of food safety knowledge, but those who were employed on a contractual basis (AOR = 0.08, 95% CI 0.01–0.49) were less likely to have a good level of food safety knowledge compared to their counterparts (Table 4).

### Food safety practices and their associated factors among meat handlers

Assessment of food safety practice of meat handlers is presented in Table 3. Nearly half of the respondents (~ 46–48%) reported that they sometimes ate, drank, or smoked in meat processing areas. The majority of them reported that they never or rarely wore an apron (96.4%) or used a hair cover (80.0%) during work. More than half of the respondents (55.7%) reported that they sometimes handled meat when they had an illness (Table 3).

**Table 3:** Summary of questions and responses for assessment of food safety practice of meat handlers working at butcher shops in Bangladesh (*N* = 300)

Questions	Responses, *n* (%)				
	Never	Rarely	Sometime	Often	Always
Do you eat or drink at your workplace?	16 (5.3)	68 (22.7)	146 (48.6)	65 (21.7)	5 (1.7)
Do you smoke inside meat processing areas?	59 (19.7)	19 (6.3)	138 (46.0)	64 (21.3)	20 (6.7)
Do you handle money while processing meat?	5 (1.7)	0 (0)	45 (15.0)	132 (44.0)	118 (39.3)
Do you wear gloves during work?	157 (52.3)	95 (31.7)	43 (14.3)	2 (0.7)	3 (1.0)
Do you wash your hands before using gloves?	209 (69.7)	51 (17.0)	7 (2.3)	2 (0.7)	31 (10.3)
Do you wear an apron during work?	287 (95.7)	2 (0.7)	3 (1.0)	2 (0.7)	6 (2.0)
Do you wear a mask during work?	149 (49.7)	99 (33.0)	47 (15.7)	2 (0.7)	3 (1.0)
Do you wear a hair cover during work?	227 (75.7)	13 (4.3)	52 (17.3)	4 (1.3)	4 (1.3)
Do you wash your hands before you touch raw meat?	103 (34.3)	16 (5.3)	81 (27.0)	76 (25.3)	24 (8.1)
Do you wash your hands after taking a break and before returning to work?	52 (17.3)	98 (32.7)	87 (29.0)	39 (13.0)	24 (8.0)
Do you wash your hands after you touch raw meat?	42 (14.0)	72 (24.0)	124 (41.3)	2 (0.7)	60 (20.0)
Do you handle/process meat when you are ill?	54 (18.0)	66 (22.0)	167 (55.7)	12 (4.0)	1 (0.3)
Do you properly clean the meat storage area before storing new products?	18 (6.0)	11 (3.7)	90 (30.0)	86 (28.7)	95 (31.6)
Do you remove your work equipment when using the toilet?	9 (3.0)	25 (8.3)	144 (48.0)	73 (24.3)	49 (16.3)
Do you handle/process meat when you have cuts, wounds, bruises, or injuries on your hands?	37 (12.3)	44 (14.7)	193 (64.3)	26 (8.7)	0 (0)

The mean food safety practice score was 23.2 (SD = 6.0, range 6–40) on a scale of 60.0. Only 16.3% (95% CI 12.3–20.7) of the respondents reported a good level of practices regarding food safety (Table 4). Chi-square tests showed that monthly family income (*p* < 0.001), number of hours worked per day (*p* < 0.001), food safety training (*p* = 0.019), and level of food safety knowledge (*p* < 0.001) were significantly associated with food safety practice (Table 5).

**Table 4:** Factors associated with the food safety knowledge of meat handlers working at butcher shops in Bangladesh (*N* = 300)

Variables	Food safety knowledge level		***p*** value	Odds ratio (95% CI)		VIF
	Good	Poor		Unadjusted	Adjusted^b^	
**Age (years)**						1.582
< 20	4 (16.7)	20 (83.3)		Reference	Reference	
21–30	22 (19.3)	92 (80.7)	0.617	1.20 (0.37–3.85)	0.80 (0.19–3.29)	
31–40	22 (18.6)	96 (81.4)		1.15 (0.36–3.69)	0.19 (0.19–3.84)	
> 40	12 (27.3)	32 (72.7)		1.86 (0.53–6.62)	1.30 (0.23–7.21)	
**Field of duty**						1.218
Butcher	35 (21.9)	125 (78.1)	0.385	Reference	Reference	
Helper	25 (17.9)	115 (82.1)		0.78 (0.44–1.38)	1.57 (0.65–3.81)	
**Education level**						2.432
No formal education	8 (12.1)	58 (87.9)		Reference	Reference	
Primary	22 (18.8)	95 (81.2)	0.101	1.68 (0.70–4.02)	2.36 (0.71–7.80)	
Secondary	18 (22.8)	61 (77.2)		2.14 (0.86–5.30)	1.86 (0.52–6.63)	
Higher secondary	12 (31.6)	26 (68.4)		3.35 (1.22–9.16)*	4.57 (1.11–18.76)*	
**Monthly earned, BDT**^a^						1.689
< 10,000	9 (15.3)	50 (84.7)		Reference	Reference	
10,001–15,000	14 (14.6)	82 (85.4)		0.95 (0.38–2.35)	0.71 (0.25–2.0)	
15,001–20,000	5 (8.3)	55 (91.7)	0.000	0.51 (0.16–1.61)	0.54 (0.14–2.08)	
20,001–25,000	6 (15.4)	33 (84.6)		1.01 (0.33–3.10)	0.90 (0.25–3.27)	
> 25,000	26 (56.5)	20 (43.5)		7.22 (2.88–18.09)***	10.52 (3.43–22.26)***	
**Work experience**						1.373
< 5 years	24 (17.1)	116 (82.9)		Reference	Reference	
5–10 years	18 (20.0)	72 (80.0)	0.343	1.21 (0.61–2.14)	0.511 (0.16–1.60)	
> 10 years	18 (15.7)	52 (74.3)		1.67 (0.84–3.35)	9.31 (1.92–15.09)**	
**Working hour/day**						1.082
< 8 h	17 (10.4)	146 (89.6)	0.000	Reference	Reference	
≥ 8 h	43 (31.4)	94 (68.6)		3.93 (2.12–7.29)***	6.14 (2.69–13.10)***	
**Employment status**						1.274
Permanent	33 (19.5)	136 (80.5)		Reference	Reference	
Daily basis	15 (21.1)	56 (78.9)	0.961	1.10 (0.56–2.19)	4.05 (1.16–14.14)*	
Contractual	12 (20.0)	48 (80.0)		1.03 (0.49–2.16)	0.08 (0.01–0.49)**	
**Food safety training**						1.056
Yes	10 (62.5)	6 (37.5)	0.000	7.80 (2.71–22.45)***	8.98 (2.16–27.32)**	
No	50 (17.6)	134 (82.4)		Reference	Reference	

*CI* confidence interval, *VIF* variance inflation factor

*p < 0.05, **p < 0.01, ***p < 0.001

^a^10,000 Bangladeshi Taka (BDT) equals to 125 USD

^b^ In this model, all the variables included in the unadjusted model were adjusted.

Hosmer and Lemeshow test for adjusted model: chi-square 8.972 (df = 8), *p* = 0.345

**Table 5:** Factors associated with the food safety practices among meat handlers working at butcher shops in Bangladesh (*N* = 300)

Variables	Food safety practice level		***p*** value	Odds ratio (95% CI)		VIF
	Good	Poor		Unadjusted	Adjusted^b^	
**Age (years)**						1.593
< 20	4 (16.7)	20 (83.3)		Reference	Reference	
21–30	15 (13.2)	99 (86.8)	0.482	0.76 (0.23–2.52)	0.49 (0.11–2.17)	
31–40	24 (20.3)	94 (79.7)		1.28 (0.40–4.09)	0.84 (0.18–3.20)	
> 40	6 (13.6)	38 (8.4)		0.79 (0.20–3.13)	0.31 (0.04–2.17)	
**Field of duty**						1.237
Butcher	26 (16.3)	134 (83.8)		Reference	Reference	
Helper	23 (16.4)	117 (83.6)	0.967	1.01 (0.55–1.87)	1.13 (0.42–3.06)	
**Education level**						1.751
No formal education	5 (7.6)	61 (92.4)		Reference	Reference	
Primary	18 (15.4)	99 (84.6)	0.059	2.21 (0.78–6.28)	2.46 (0.71–8.49)	
Secondary	16 (20.3)	63 (79.7)		3.02 (1.07–8.98)*	3.17 (0.81–12.47)	
Higher secondary	10 (26.3)	28 (73.7)		4.36 (1.36–13.94)*	2.48 (0.55–11.12)	
**Monthly earned, BDT**^a^						1.700
< 10,000	8 (13.6)	51 (86.4)		Reference	Reference	
10,001–15,000	12 (12.5)	84 (87.5)		0.91 (0.35–2.38)	0.85 (0.28–2.55)	
15,001–20,000	7 (11.7)	53 (88.3)	0.000	0.29 (0.29–2.50)	1.16 (0.31–4.40)	
20,001–25,000	4 (10.3)	35 (89.7)		0.73 (0.20–2.61)	0.41 (0.09–1.89)	
> 25,000	18 (39.1)	28 (60.9)		4.20 (1.58–10.62)**	2.46 (0.70–8.65)	
**Work experience**						1.379
< 5 years	20 (14.3)	120 (85.7)		Reference	Reference	
5–10 years	14 (15.6)	76 (84.4)	0.407	1.11 (0.53–2.32)	0.43 (0.12–1.52)	
> 10 years	15 (21.4)	55 (78.6)		1.64 (0.78–3.44)	0.18 (0.02–1.84)	
**Working hour/day**						1.146
< 8 h	9 (5.5)	154 (94.5)	0.000	Reference	Reference	
≥ 8 h	40 (29.2)	97 (70.8)		7.06 (3.28–15.19)***	8.44 (3.11–22.91)***	
**Employment status**						1.274
Permanent	23 (13.6)	146 (86.4)		Reference	Reference	
Daily basis	11 (15.5)	60 (84.5)	0.119	1.16 (0.53–2.54)	3.73 (0.93–14.10)	
Contractual	15 (25.0)	45 (75.0)		2.12 (1.02–4.40)*	10.86 (0.93–27.10)	
**Food safety training**						1.116
Yes	6 (37.5)	10 (62.5)	0.019	3.36 (1.16–9.73)*	1.29 (0.29–5.74)	
No	43 (15.1)	251 (83.7)		Reference	Reference	
**Food safety knowledge**						1.172
Good	28 (46.7)	32 (53.3)	0.000	9.13 (4.64–17.95)***	5.68 (2.33–13.87)***	
Poor	21 (8.8)	219 (91.3)		Reference	Reference	

*CI* confidence interval, *VIF* variance inflation factor

*p < 0.05, **p < 0.01, ***p < 0.001

^a^10,000 Bangladeshi Taka (BDT) equals to 125 USD

^b^ In this model, all the variables included in the unadjusted model were adjusted

Hosmer and Lemeshow test for adjusted model: chi-square: 11.674 (df = 8), *p* = 0.166

Table 5 depicts the findings from the multiple logistic regression analyses. Adjusted regression analyses demonstrated that the odds of having a good level of practice was 8.5 times higher among respondents who worked for ≥ 8 h per day compared to those who worked < 8 h per day (AOR = 8.44, 95% CI 3.11–22.91). The level of food safety practice was 5.7 times higher among respondents who had a good level of food safety knowledge compared to their counterparts (AOR = 5.68, 95% CI 2.33–13.87).

## Discussion

This study assessed the factors associated with food safety knowledge and practices among meat handlers in butcher shops in Bangladesh. Overall, the food safety knowledge and practice among meat handlers in this study was low, at 20% and 16.3%, respectively. Nevertheless, within the individual constructs, the respondents were more knowledgeable about some food safety issues than others. For instance, the majority of the respondents knew that handwashing before work, using gloves, caps and aprons, and proper cleaning of the instruments reduces the risk of food contamination. However, they were less knowledgeable about high-risk groups for food poisoning as well as selected foodborne diseases and foodborne pathogens. This is consistent with previous studies that have found meat handlers to be more knowledgeable about handwashing and use of protective equipment compared to other domains of food safety [4, 18, 40].

Concerning factors associated with food safety knowledge, our findings revealed that education, work experience, training, and income were significantly associated with food safety knowledge. Specifically, meat handlers who had higher secondary education and received some form of training on food safety reported greater likelihood of having high knowledge of food safety. The result is corroborated by a related study conducted in South Africa [18] that showed that educational level and professional training of meat handlers was significantly associated with their level of knowledge and food safety practices. Plausibly, through education and professional training, meat handlers are exposed to food safety issues such as handwashing, use of gloves, caps and aprons, and proper cleaning of the instruments as well as identifying the pathways through will they can contaminate meat during the handling process. Hence, this may explain why those with higher education and training had higher food safety knowledge. This finding highlights the importance of promoting higher education in Bangladesh and also emphasizes the need to provide regular training for meat handlers. As indicated by a recent study conducted in Bangladesh [27], the majority (85%) of meat handlers in slaughter houses and meat selling centers reported that they did not receive any form of training concerning food safety and meat hygiene; however, approximately 90% of meat handlers reported they were willing to undergo training on food safety and meat hygiene. Given the absence of training programs for meat handlers in Bangladesh, it is imperative to provide such training concerning food safety and meat hygiene to all meat handlers.

Meat handlers who reported a higher income and had worked for more than 10 years were more likely to have higher food safety knowledge. This could be explained by the notion that meat handlers who receive higher income are able to afford professional training on food safety, thereby translating into higher food safety knowledge as observed in this study. However, this finding is contrary to a related study by Yenealem, Yallew, and Abdulmajid [41] that found there was no significant association between years of experience and food safety knowledge, or income level and food safety knowledge.

It is indicative from our findings that high food safety knowledge could translate into good food safety practice. Essentially, this study shows that respondents with food safety knowledge were 5.7 times more likely to engage in food safety practices. Our findings also show a higher association between food safety knowledge and food safety practice, which is in agreement with other recent findings from a study by Yenealem, Yallew, and Abdulmajid [41] that found meat handlers with a high level of food safety knowledge were 2 times more likely to be engaged in good meat handling practices. Further, the results from our study are in agreement with previous studies conducted among food handlers from Ethiopia [36], Romania [19], and Saudi Arabia [28]. These findings imply that food safety knowledge could be an essential component to the promotion of good food handling practices (e.g., hygienic meat handling).

### Strengths and limitations

The strength of our study lies in its analytical rigor and detailed methodological approaches. Moreover, this is one of the first studies to examine the factors affecting food safety knowledge and practices among meat handlers in a region that has several foodborne illnesses every year. Our findings may provide baseline data for future interventions to improve food safety knowledge and practices. However, this study was not without limitations which should be considered in the interpretation of our findings. This study employed a cross-sectional research design which limits the establishment of causal pathways. Due to the specific areas of Bangladesh explored in this study (i.e., four districts from coastal regions), findings may not generalized to the whole country. Food safety practices were assessed by information provided by the respondents instead of observational checklists which may lead to reporting bias. However, we used a 5-point Likert scale for assessing food safety practices which may reduce reporting bias.

## Conclusions

In summary, meat handlers in Bangladesh have low food safety knowledge and practices, and thus, there is a need for interventions to improve their knowledge regarding food safety. Findings showed that higher secondary education, greater work experience, working hours per day, training, and higher income are factors associated with better food safety knowledge among meat handlers in Bangladesh. Health department officials need to establish and augment existing mechanisms that aim to increase the proportion of persons with higher education in the country. Butcher shops in Bangladesh could consider the hiring of employees with higher level of education in order to ensure higher safety measures are practiced in these facilities. The findings also emphasize the need for butcher shops and government agencies in charge of health promotion to engage in regular training for meat handlers. This is imperative to improve their food safety knowledge and can potentially improve meat handling practices. This training program must be formalized with clear guidelines that address food safety and meat hygiene issues in order to better educate meat handlers. This would require the Bangladesh food safety authority (BFSA) to include provisions for regular training sessions for meat handlers as part of their core mandates of regulating and coordinating the meat handling industry. This could be done by moving through the various meat handling facilities and organizing short but comprehensive training for meat handlers. Further, in order to make the trainings effective, it is imperative for the BFSA to make undertaking of regular training a mandatory requirement for licensing meat handlers. Also, efforts would have to be made to facilitate the endorsement of HACCP at all levels of meat handling in order to ensure adherence to food safety measures. Our findings also have some bearing on Bangladesh’s capacity to attain Sustainable Development Goals (SDG) 2.2 by 2030. Lastly, there is a need for intervention and longitudinal studies that incorporate large, diverse samples of Bangladeshi meat handlers to determine factors related to their knowledge and practices regarding food safety in order to minimize foodborne illnesses and diseases in Bangladesh.

## Data Availability

The datasets for this study are available from the corresponding author on reasonable request.

## Abbreviations

WASH: Water, Sanitation and Hygiene
IEDCR: Institute of Epidemiology, Disease Control and Research
HACCP: Hazard Analysis and Critical Control Point
BSTI: Bangladesh Standards and Testing Institution
ISO: International Standard Organization
SPSS: Statistical Package for the Social Sciences
BDT: Bangladeshi taka
USD: United States dollar
CI: Confidence interval
AOR: Adjusted odds ratio
BFSA: Bangladesh Food Safety Authority
SDG: Sustainable Development Goals
